# Flow diverter treatment of posterior circulation aneurysms. A meta-analysis

**DOI:** 10.1007/s00234-016-1649-2

**Published:** 2016-01-22

**Authors:** Cheng-Bin Wang, Wen-Wen Shi, Guang-Xu Zhang, Hu-Chen Lu, Jun Ma

**Affiliations:** Department of Neurosurgery, Nanjing Brain Hospital Affiliated to Nanjing Medical University, Nanjing, No. 264, Guangzhou Road, Nanjing, Jiangsu 210000 China; School of Inspection and Life Science, Wenzhou Medical University, Wenzhou, Zhejiang China

**Keywords:** Endovascular treatment, Flow diverters, Interventional neuroradiology, Posterior circulation aneurysms

## Abstract

**Introduction:**

Treatment of complex anterior circulation aneurysms with flow diverters (FDs) has become common practice in neurovascular centers. However, this treatment method for posterior circulation aneurysms (PCAs) still remains controversial.

**Methods:**

Through searches for reports on the treatment of PCAs with FDs, we conducted a systematic review of the literature on its clinical efficacy and safety using random-effect binomial meta-analysis.

**Results:**

We included 14 studies, which reported on a total of 225 PCAs in 220 patients. Procedure-related good outcome rate was 79 % (95 % confidence interval (CI), 72–84), with significantly lower odds among patients with ruptured aneurysms and basilar artery aneurysms. Procedure-related mortality rate was 15 % (95 % CI 10–21), with significantly higher rates among patients with giant aneurysms and basilar artery aneurysms. The rate of complete aneurysm occlusion at 6-month digital subtraction angiography (DSA) was 84 %. Ischemic stroke rate was 11 %. Perforator infarction rate was 7 %. Postoperative subarachnoid hemorrhage (SAH) rate was 3 %. Intraparenchymal hemorrhage (IPH) rate was 4 %.

**Conclusions:**

Flow diverter treatment of PCAs is an effective method, which provides a high rate of complete occlusion at 6-month DSA. However, compared with anterior circulation aneurysms, patients with PCAs are at significantly higher risk of mortality, ischemic stroke and perforator infarction. Our findings indicate that, in most clinical centers, flow diverter treatment of PCAs should be conducted in carefully selected patients with poor natural history and no optimal treatment strategy. For ruptured and giant basilar artery aneurysms, there is still no good treatment option.

Flow diverters (FDs), as an addition to the endovascular treatment options for intracranial aneurysms, have higher surface coverage and lower porosity than conventional intracranial stents. Their design focuses on diverting flow from the aneurysm, thus creating an environment prone to thrombosis. FDs may also provide scaffolding for endothelialization and vessel wall healing. Treatment of complex anterior circulation aneurysms, predominantly internal carotid artery (ICA) aneurysms, with FDs has become common practice in neurovascular centers [1–3]. However, flow diverter treatment for posterior circulation aneurysms (PCAs) still remains controversial because of significantly higher rates of ischemic stroke and perforator infarction than those observed for anterior circulation aneurysms [1–4].

In recent years, several studies have reported on the safety and efficacy of flow diverter treatment for PCAs [1–3, 5–15], but none have systematically evaluated the rate of mortality, aneurysmal occlusion, and procedure-related complications. It is therefore the aim of this systematic review and meta-analysis to provide a systematic understanding of the flow diverter treatment for PCAs, which would help guide practitioners in selecting the best therapy method for patients with complex PCAs.

## Methods

We conducted PubMed, OVID, and Web of Science searches to review all studies on the treatment of PCAs with FDs. In brief, we used the keywords “Posterior Circulation Aneurysms,” “PED,” “pipeline embolization device,” “flow diverter,” “divert,” “diversion,” and “pipeline.” Inclusion criteria were as follows: English language, ≥5 patients, studies published between July 2005 and July 2015, and the presence of data on procedure-related good outcome or mortality or aneurysmal occlusion rate or postoperative complications. The exclusion criteria were as follows: case reports, in vitro or cadaveric studies, review articles, guidelines, technical notes, and whether only a subset of patients of the total number of patients treated with FDs was analyzed. We also searched the reference lists of all eligible studies and pertinent publications for additional studies.

In the case of overlapping study populations, we tried to exclude the possibility of individual patients being described twice. When patients were included in multiple studies, we contacted the study authors by e-mail to exclude the duplicate cases. If the repetitive data could not be definitively sorted out, the report with the higher number of patients and/or the longer follow-up was selected [16]. Abstracts, methods, results, figures, and tables of the full studies were searched for data on procedure-related good outcome and mortality, aneurysmal occlusion rates, and postoperative complications.

Epidemiological data included the number of patients and aneurysms, the features of the aneurysms as well as the clinical presentation (incidental, hemorrhagic, or symptomatic) of the patients. Aneurysms were classified as small (<10 mm), large (10 mm ≤ aneurysm size ≤ 25 mm), or giant (>25 mm). The shape of aneurysms was classified as saccular and not saccular. The state of aneurysms was divided into ruptured and unruptured, herein ruptured aneurysms referred to the ones received flow diverter treatment in the acute stage. The term good outcome was defined as modified Rankin scale of 0–2 (mRS 0–2) and mortality as mRS 6. Aneurysmal occlusion was defined as complete occlusion at 6-month digital subtraction angiography (DSA). Postoperative complications were analyzed with respect to ischemic stroke, perforator infarction, subarachnoid hemorrhage (SAH), and intraparenchymal hemorrhage (IPH). Subgroup analysis was conducted between study outcomes and the characteristics of aneurysms and patients.

### Statistical analyses

Random-effect meta-analysis was performed on studies that provided data on outcomes of patients who underwent flow diverter treatment. We estimated from each study the cumulative incidence (event rate) and 95 % CI for each outcome. Subgroup analysis was carried out to evaluate the impact of the preoperative conditions on the results, which was presented as odds ratios (OR) with a 95 % CI. Statistical heterogeneity across studies was assessed using the *I*^2^ statistic. We evaluated potential publication bias by using funnel plots.

## Results

### Study selection

**Fig. 1:**
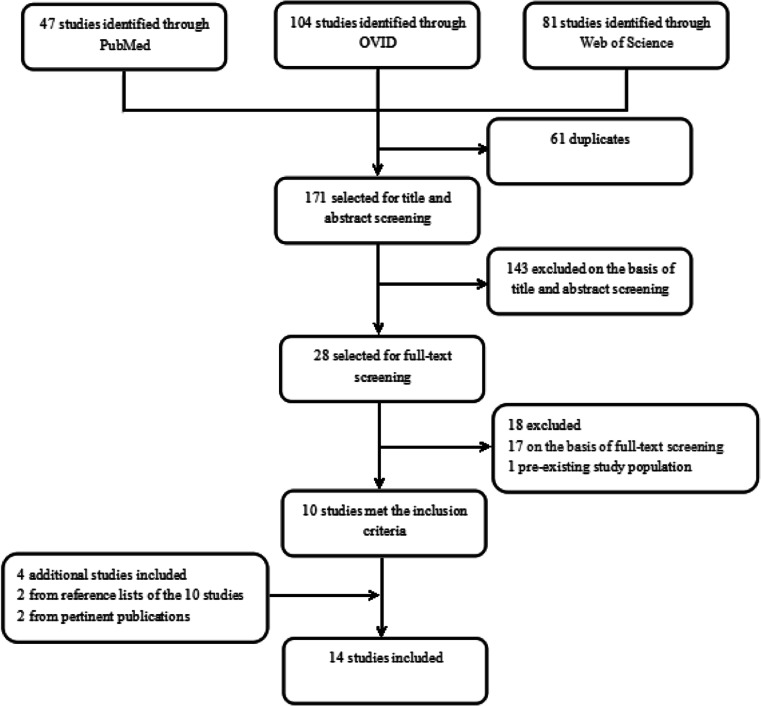
Selection of included studies

Our search strategy revealed a total number of 171 different studies, 143 of which were excluded by title and abstract screening. Of the 28 remaining studies, full texts were accessed, and ten studies met our predefined inclusion criteria. Two additional studies were found by considering the reference lists of the ten previously mentioned studies, and another two additional studies were found from pertinent publications. As a result, a total of 14 studies were included in the analysis (Table 1), including seven prospective single-arm cohort studies and seven retrospective case series. Figure 1 presents a flow chart illustrating the above search process.

**Table 1:** Studies included in meta-analysis

Author	Title	Journal	Year	Study design	No. of patients	Aneurysms treated
Byrne, J.V. et al. [5]	Early experience in the treatment of intracranial aneurysms by endovascular flow diversion: a multicenter prospective study	PLoS ONE	2010	Prospective	20	20
Kulcsar, Z. et al. [9]	High-profile flow diverter (silk) implantation in the basilar artery: efficacy in the treatment of aneurysms and the role of the perforators	Stroke	2010	Retrospective	12	12
Chalouhi, N. et al. [3]	Treatment of posterior circulation aneurysms with the pipeline embolization device	Neurosurgery	2013	retrospective	7	7
Siddiqui, A.H. et al. [12]	Panacea or problem: flow diverters in the treatment of symptomatic large or giant fusiform vertebrobasilar aneurysms	Neurosurgery	2012	Prospective	7	7
Toth, G. et al. [14]	Posterior circulation flow diversion: a single-center experience and literature review	Journal of Neurointerventional Surgery	2015	Retrospective	6	7
Meckel, S. et al. [7]	Endovascular treatment of complex aneurysms at the vertebrobasilar junction with flow-diverting stents: initial experience	Neurosurgery	2013	Retrospective	7	7
Toma, A.K. et al. [6]	Early single centre experience of flow diverting stents for the treatment of cerebral aneurysms	British Journal of Neurosurgery	2013	Prospective	17	17
Monteith, S.J. et al. [8]	Endovascular treatment of fusiform cerebral aneurysms with the pipeline embolization device	Neurosurgery	2014	Retrospective	7	7
Phillips, T.J. et al. [2]	Safety of the pipeline embolization device in treatment of posterior circulation aneurysms	American Journal of Neuroradiology	2012	Prospective	32	32
Munich, S.A., et al. [13]	The pipeline embolization device for the treatment of posterior circulation fusiform aneurysms: lessons learned at a single institution	Neurosurgery	2014	retrospective	12	12
McAuliffe, W. et al. [10]	Immediate and midterm results following treatment of recently ruptured intracranial aneurysms with the pipeline embolization device	American Journal of Neuroradiology	2012	Prospective	6	6
De Vries, J. et al. [11]	New generation of Flow Diverter (surpass) for unruptured intracranial aneurysms: a prospective single-center study in 37 patients	Stroke	2013	Prospective	5	5
Kallmes, D.F. et al. [1]	International retrospective study of the pipeline embolization device: a multicenter aneurysm treatment study	American Journal of Neuroradiology	2015	Retrospective	55	59
Wakhloo, A.K. et al. [15]	Surpass flow diverter in the treatment of intracranial aneurysms: a prospective multicenter study	American Journal of Neuroradiology	2015	Prospective	27	27

### Aneurysm and patient characteristics

**Fig. 2:**
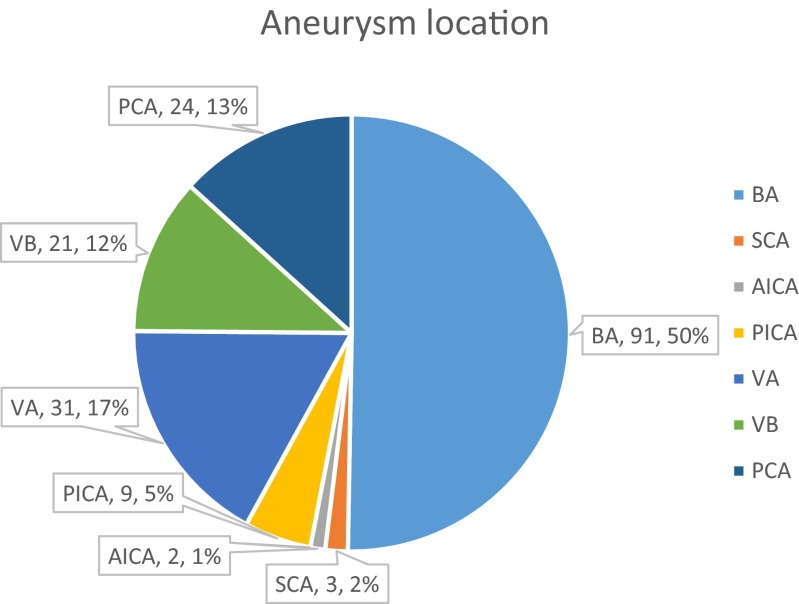
The location distribution of the aneurysms. *BA* basilar artery, *SCA* superior cerebellar artery, *AICA* anterior inferior cerebellar artery, *PICA* posterior inferior cerebellar artery, *VA* vertebral artery, *VB* vertebrobasilar junction, *PCA* posterior cerebral artery

A total of 220 patients with 225 PCAs (16 % [*n* = 25, 95 % CI 10–22] ruptured, 84 % [*n* = 136, 95 % CI 78–90] unruptured) were included in the analyses. In terms of size, 29 % of the aneurysms were classed as small (*n* = 48, 95 % CI 22–37), 48 % (*n* = 79, 95 % CI 40–56) were classed as large, and 23 % (*n* = 38, 95 % CI 17–30) were giant aneurysms. Thirty-four percent (*n* = 58, 95 % CI 27–42) were saccular aneurysms, and 66 % (*n* = 111, 95 % CI 58–73) were not; 26 % (*n* = 27, 95 % CI 18–36) of the patients with aneurysms received FD treatment as a retreatment. Of the 220 patients, 61 % (*n* = 62, 95 % CI 51–70) were symptomatic, and the other 39 % (*n* = 40, 95 % CI 30–49) were asymptomatic (Table 2). The location distribution of the aneurysms was showed in Fig. 2.

**Table 2:** Characteristics of aneurysms and patients

	Rate	95 % CI	*N*
Posterior circulation aneurysm			
Small	0.29	0.22–0.37	48
Large	0.48	0.40–0.56	79
Giant	0.23	0.17–0.30	38
Saccular	0.34	0.27–0.42	58
Not saccular	0.66	0.58–0.73	111
Ruptured	0.16	0.10–0.22	25
Unruptured	0.84	0.78–0.90	136
Retreatment	0.26	0.18–0.36	27
First treatment	0.74	0.64–0.82	75
Patient			
Symptomatic	0.61	0.51–0.70	62
Asymptomatic	0.39	0.3–0.49	40

*CI* confidence interval, *N* number

### Study outcomes

**Fig. 3:**
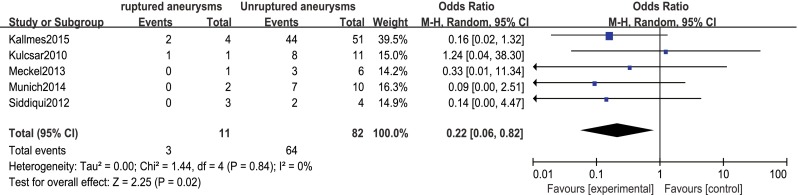
Forest plot and meta-analysis of procedure-related good outcome rate: ruptured aneurysms vs. unruptured aneurysms. *M-H* Mantel-Haenszel method, *CI* confidence interval

**Fig. 4:**
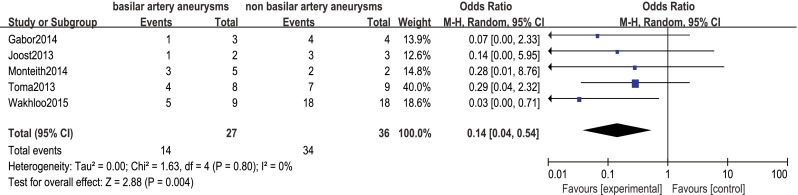
Forest plot and meta-analysis of procedure-related good outcome rate: basilar artery aneurysms vs. non basilar artery aneurysms. *M-H* Mantel-Haenszel method, *CI* confidence interval

Procedure-related good outcome rate was 79 % (95 % CI 72–84), with significantly lower rates among patients with ruptured aneurysms and basilar artery aneurysms (OR 0.22, 95 % CI 0.06–0.82 and OR 0.14, 95 % CI 0.04–0.54, respectively; Figs. 3 and 4). Aneurysm size, aneurysm type, preoperative symptoms, and prior treatment were not significantly associated with the rate of procedure-related good outcome (OR 0.99, 95 % CI 0.37–2.66; OR 6.20, 95 % CI 0.69–55.55; OR 0.20, 95 % CI 0.04–1.07; and OR 1.72, 95 % CI 0.40–7.34, respectively).

**Fig. 5:**
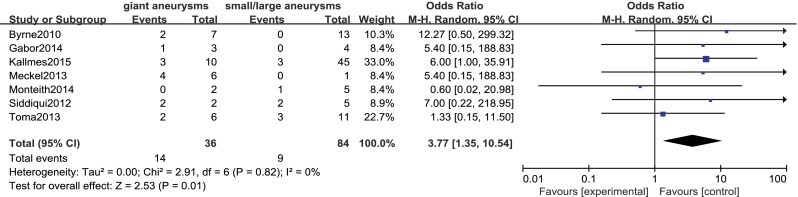
Forest plot and meta-analysis of procedure-related mortality rate: giant aneurysms vs. small/large aneurysms. *M-H* Mantel-Haenszel method, *CI* confidence interval

**Fig. 6:**
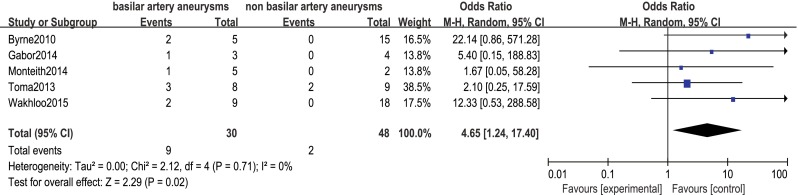
Forest plot and meta-analysis of procedure-related mortality rate: basilar artery aneurysms vs. non basilar artery aneurysms. *M-H* Mantel-Haenszel method, *CI* confidence interval

Procedure-related mortality rate was 15 % (95 % CI 10–21), with significantly higher rates among patients with giant aneurysms and basilar artery aneurysms (OR 3.77, 95 % CI 1.35–10.54 and OR 4.65, 95 % CI 1.24–17.40, respectively; Figs. 5 and 6). Aneurysm state, aneurysm type, preoperative symptoms, and prior treatment were not significantly associated with the rate of procedure-related mortality (OR 1.96, 95 % CI 0.28–13.62; OR 0.15, 95 % CI 0.02–1.13; OR 2.17, 95 % CI 0.17–27.91; and OR 0.33, 95 % CI 0.04–2.55, respectively).

The rate of aneurysm complete occlusion at 6-month DSA was 84 % (95 % CI 68–94), with no significant association with aneurysm size (OR 3.60, 95 % CI 0.26–50.84). Ischemic stroke rate was 11 % (95 % CI 7–17) with no significant association with aneurysm size (OR 1.06, 95 % CI 0.26–4.30). Perforator infarction rate was 7 % (95 % CI 3–13). Postoperative SAH was 3 % (95 % CI 1–6 %). IPH rate was 4 % (95 % CI 1–8). The above data are summarized in Table 3.

**Table 3:** Outcomes for endovascular treatment of posterior circulation aneurysms with FDs

Outcome		Rate (95 % CI)	OR	95 % CI	*I* ^2^ (%)
Procedure-related mortality (mRS 6)		0.15 (0.10–0.21)			
Aneurysm size (giant vs. small/large)^a^	Giant	0.37 (0.22–0.54)	3.77	1.35–10.54	0
	Small/large	0.08 (0.04–0.15)			
Aneurysm type (saccular vs. not saccular)	Saccular	0 (0–0.12)	0.15	0.02–1.13	0
	Not saccular	0.18 (0.10–0.28)			
Aneurysm location (basilar artery vs. not basilar artery)^a^	Basilar artery	0.25 (0.14–0.37)	4.65	1.24–17.40	0
	Not basilar artery	0.07 (0.02–0.18)			
Aneurysm state (rupture vs. unrupture)	Rupture	0.14 (0.03–0.36)	1.96	0.28–13.62	0
	Unrupture	0.11 (0.05–0.20)			
Preoperative symptoms (with vs. without)	With	0.18 (0.10–0.30)	2.17	0.17–27.91	0
	Without	0 (0–0.10)			
Prior treatment (retreatment vs. first treatment)	Retreatment	0 (0–0.14)	0.33	0.04–2.55	0
	First treatment	0.16 (0.08–0.26)			
Procedure-related good outcome (mRS 0–2)		0.79 (0.72–0.84)			
Aneurysm size (small vs. large/giant)	Small	0.73 (0.57–0.86)	0.99	0.37–2.66	11
	Large/giant	0.70 (0.60–0.79)			
Aneurysm type (saccular vs. not saccular)	Saccular	1 (0.83–1)	6.2	0.69–55.55	0
	Not saccular	0.70 (0.57–0.81)			
Aneurysm location (basilar artery vs. not basilar artery)^a^	Basilar artery	0.55 (0.41–0.69)	0.14	0.04–0.54	0
	Not basilar artery	0.95 (0.83–0.99)			
Aneurysm state (rupture vs. unrupture)^a^	Rupture	0.68 (0.46–0.85)	0.22	0.06–0.82	0
	Unrupture	0.82 (0.74–0.88)			
Preoperative symptoms (with vs. without)	With	0.65 (0.52–0.77)	0.2	0.04–1.07	0
	Without	0.97 (0.85–1)			
Prior treatment (retreatment vs. first treatment)	Retreatment	0.92 (0.73–0.99)	1.72	0.40–7.34	0
	First treatment	0.73 (0.61–0.83)			
Complete occlusion rate at 6-month DSA		0.84 (0.68–0.94)			
Aneurysm size (small vs. large/giant)	Small	1 (0.40–1)	3.6	0.26–50.84	0
	Large/giant	0.6 (0.15–0.95)			
Ischemic stroke		0.11 (0.07–0.17)			
Aneurysm size (small vs. large/giant)	Small	0.13 (0.04–0.29)	1.06	0.26–4.30	0
	Large/giant	0.14 (0.07–0.24)			
Perforator infarction		0.07 (0.03–0.13)			
Subarachnoid hemorrhage		0.03 (0.01–0.06)			
Intraparenchymal hemorrhage		0.04 (0.01–0.08)			

*CI* confidence interval, *mRS* modified Rankin scale, *DSA* digital subtraction angiography, *OR* odds ratio

^a^Denotes statistically significant results

### Sensitivity analysis and publication bias

**Fig. 7:**
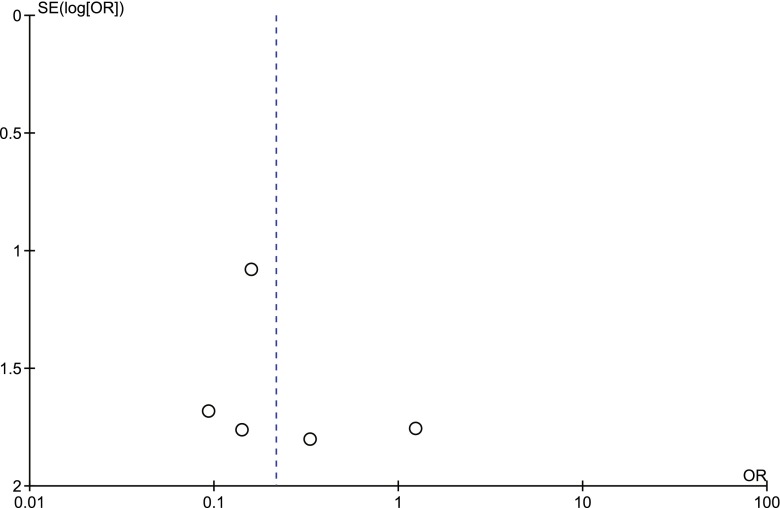
Procedure-related good outcome rate: ruptured aneurysms vs. unruptured aneurysms. *SE* standard error, *OR* odds ratio

**Fig. 8:**
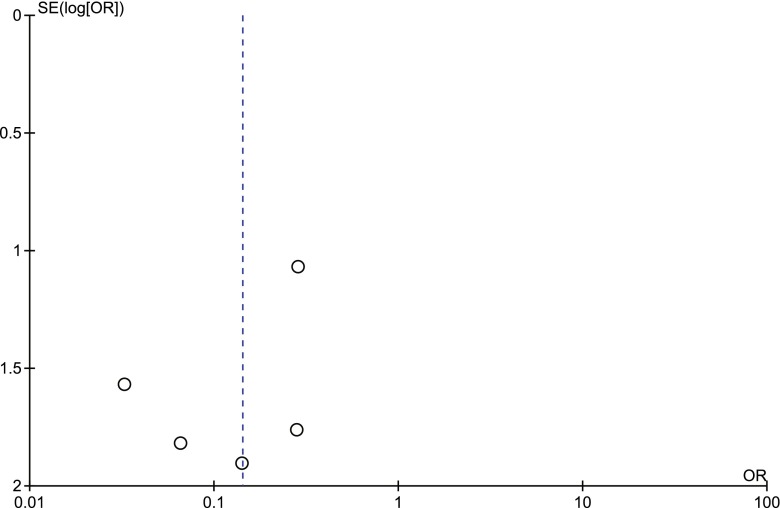
Procedure-related good outcome rate: basilar artery aneurysms vs. non basilar artery aneurysms. *SE* standard error, *OR* odds ratio

**Fig. 9:**
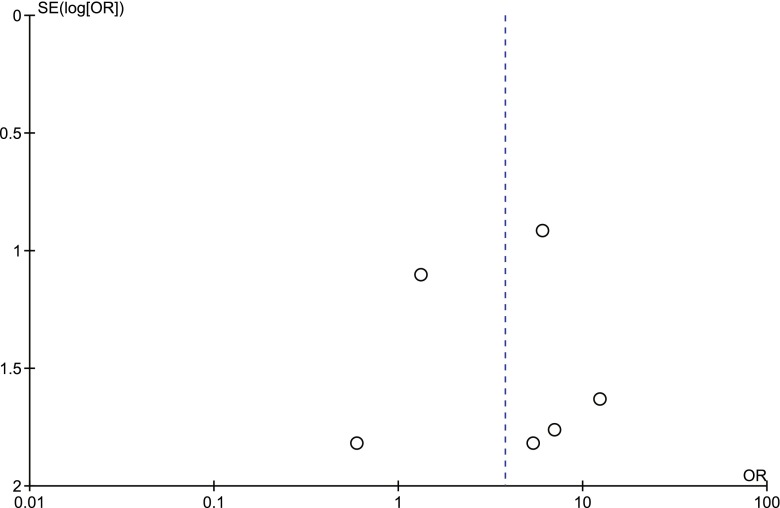
Procedure-related mortality rate: giant aneurysms vs. small/large aneurysms. *SE* standard error, *OR* odds ratio

**Fig. 10:**
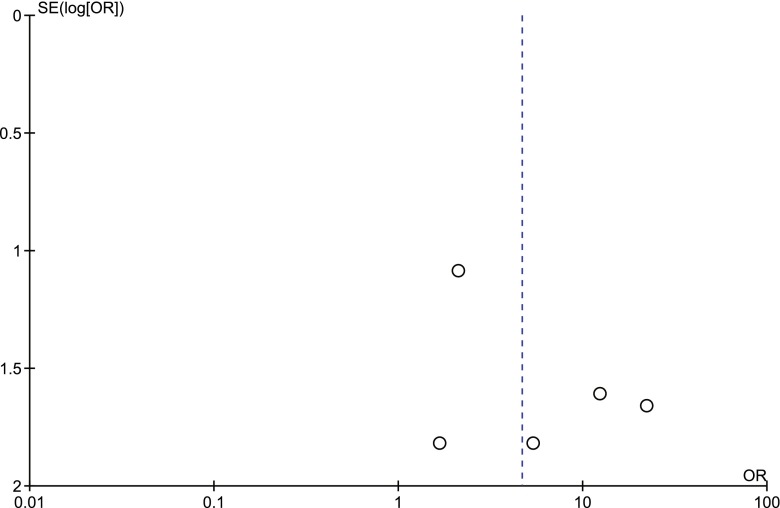
Procedure-related mortality rate: basilar artery aneurysms vs. non basilar artery aneurysms. *SE* standard error, *OR* odds ratio

None of the analyses conducted exhibited heterogeneity except for the analysis of procedure-related good outcome with different size aneurysms (*I*^2^ = 11 %), suggesting unexplained minimal differences in study populations and procedures. Funnel plot analyses on the statistically significant studies are shown in Figs. 7, 8, 9, and 10, which indicated significant publication bias.

## Discussion

With recent advancements in neuroendovascular technology, flow-diverting stents provided a new therapeutic option of total intraluminal reconstruction for the treatment of intracranial aneurysm. But compared with traditional clipping and coiling, the use of FDs was restricted to giant and complex aneurysms. Our meta-analysis included 225 aneurysms. Large or giant aneurysms accounted for 71 % of the total, and 66 % of the aneurysms were classified as not saccular aneurysms, including fusiform, dissecting, blister, and other complex aneurysms. Similar to conventional stents, flow-diverting stents have also been controversial in the treatment of ruptured aneurysms because of the necessity of antiaggregation pre- and post-operation. Of the 225 aneurysms reported here, 84 % were unruptured. For ruptured aneurysms, there is still no evidence-based concept or large agreement on antiplatelet and anticoagulation schedule. Stephan et al. [7] gave patients with ruptured aneurysms an intravenous bolus of heparin (5000 IU) and aspirin (500 mg) at the start of the procedure. After the procedure, intravenous heparin continued for 2 days; double antiplatelet therapy with clopidogrel (75–150 mg/day) and aspirin (81–325 mg/day) was continued for variable duration. However, in other clinical centers, patients received a loading dose of only 300 mg clopidogrel and 300 mg aspirin 6 h before aneurysm treatment [9], a loading dose of clopidogrel 600 mg and aspirin 325 mg the night before surgery [13], or 300/600 mg of clopidogrel on the day of the procedure [2].

In our meta-analysis, 79 % of the patients showed a good outcome. The rates ranged from 29 to 100 % [1, 2, 6–15], highlighted by significantly lower rates in the patients with ruptured and basilar artery aneurysms. Meanwhile, patients with basilar artery aneurysms had a higher mortality rate. The basilar artery is rich of perforator arteries, most of which supply the cerebellum, brain stem, and other important structures. What is worse, these areas lack effective vascular compensatory mechanisms. As a consequence, a relatively higher perforator infarction rate was observed when FDs were placed in the vascular lumen. The association between the location of the aneurysms and the rate of perforator infarction during the follow-up was not analyzed in our meta-analysis because of a lack of information in many studies, but studies reporting the perforator infarction of basilar artery reported relatively higher rates from 14 to 25 % [2, 7–9]. These results may account for adverse outcomes in patients with basilar artery aneurysms. Patients with ruptured aneurysms had worse preoperative status compared to those with unruptured aneurysms. These factors likely lead to a lower good outcome rate, but not to a higher mortality rate.

Mortality rate was another important indicator for evaluating the safety of flow diverter treatment for patients with PCAs. Published mortality rates were variable, ranging from 0 to 57 % [1, 2, 5–15]. Our meta-analysis provided more representative data on mortality rate, with significantly higher rates among patients with giant and basilar artery aneurysms. What accompanying with giant aneurysms are always rupture, preoperative symptoms, higher rates of ischemic stroke, and postoperative SAH [4]; none of which are conducive to the recovery of patients. However, patients with ruptured aneurysms or preoperative symptoms did not have a higher mortality rate than patients without. In the meta-analysis reported by Brinjikji et al. [4], 1451 patients with 1654 intracranial aneurysms were treated with FDs, and the total mortality rate was 4 %, which was significantly lower than that in the patients with PCAs reported here.

The target of endovascular treatment is to prevent aneurysms from either the first or a repeated rupture, so the occlusion rate is the most important indicator to measure the effectiveness of flow diverter treatment. We found a complete occlusion rate of more than 80 % at 6-month DSA, which compared favorably with that of stent-assisted [17] or balloon-assisted embolization [18].

The main complications of FDs are ischemic stroke, perforator infarction, postoperative SAH, and IPH. They were not rare in our meta-analysis, as only one study definitively reported none of these complications [3]. Ischemic stroke was the most common complication, followed by perforator infarction and IPH, and postoperative SAH. Among these complications, the rates of ischemic stroke and perforator infarction were apparently higher than those reported for flow diverter treatment of intracranial aneurysms, which were 6 and 3 % respectively [4]. High ischemic complications may relate with the lack of optimal platelet inhibition, so platelet function tests should be performed on all patients prior to the procedure to make sure that the level of platelet inhibition was adequate (>30 %) [3, 8]. What is more, adverse event rates drop significantly with experience. Brinjikji et al. [4] reported a significantly higher rate of ischemic stroke among patients with large/giant aneurysms, and ascribed the cause to the longer operation time. We also analyzed the association between the ischemic stroke rate and the size of the aneurysms, but did not find similar results. This may be partly due to the small number of cases analyzed.

Several limitations might have affected our results. Publication bias is the most common systematic error of meta-analysis, and it should be carefully considered here because our results were based mostly on small studies. Compared with large studies, small studies have reported more adverse outcomes, and studies describing only a small number of patients may be more easily accepted for publication if they alert for any adverse events [16]. Secondly, the available studies were of poor quality, as approximately half were retrospective case series. Thirdly, because studies with significant results are more likely to be published in English, we only included English language articles. As a consequence, it is possible that some high quality studies in other languages might have been excluded. Finally, the standard of selecting patients, pre- and post-procedural antiplatelet/anticoagulation protocol, the number and kind of the stents used, and personal experience with stenting techniques varied in studies.

## Conclusions

Flow diverter treatment of PCAs is an effective method which provides a high rate of complete occlusion at 6-month DSA. But compared with treatment of anterior circulation aneurysms, it has significantly higher rates of mortality, ischemic stroke and perforator infarction. Our findings indicate that, in most clinical centers, flow diverter treatment of PCAs should be conducted in carefully selected patients with poor natural history and no optimal treatment strategy. For ruptured and giant basilar artery aneurysms, there is still no good treatment option, and the problem is unsolved. The findings reported herein suggest that further well-designed prospective large studies are needed.

## References

[1] Kallmes DF, Hanel R, Lopes D, Boccardi E, Bonafe A, Cekirge S, Fiorella D, Jabbour P, Levy E, McDougall C, Siddiqui A, Szikora I, Woo H, Albuquerque F, Bozorgchami H, Dashti SR, Delgado Almandoz JE, Kelly ME, Turner R, Woodward BK, Brinjikji W, Lanzino G, Lylyk P (2015). International retrospective study of the pipeline embolization device: a multicenter aneurysm treatment study. AJNR Am J Neuroradiol.

[2] Phillips TJ, Wenderoth JD, Phatouros CC, Rice H, Singh TP, Devilliers L, Wycoco V, Meckel S, McAuliffe W (2012). Safety of the pipeline embolization device in treatment of posterior circulation aneurysms. AJNR Am J Neuroradiol.

[3] Chalouhi N, Tjoumakaris S, Dumont AS, Gonzalez LF, Randazzo C, Starke RM, Rosenwasser RH, Jabbour P (2013). Treatment of posterior circulation aneurysms with the pipeline embolization device. Neurosurgery.

[4] Brinjikji W, Murad MH, Lanzino G, Cloft HJ, Kallmes DF (2013). Endovascular treatment of intracranial aneurysms with flow diverters: a meta-analysis. Stroke J Cereb Circ.

[5] Byrne JV, Beltechi R, Yarnold JA, Birks J, Kamran M (2010) Early experience in the treatment of intra-cranial aneurysms by endovascular flow diversion: a multicentre prospective study. PloS one 5 (9). doi:10.1371/journal.pone.0012492 10.1371/journal.pone.0012492 PMC2932685 20824070

[6] Toma AK, Robertson F, Wong K, Joshi Y, Haridas A, Grieve J, Watkins LD, Kitchen ND, Brew S (2013). Early single centre experience of flow diverting stents for the treatment of cerebral aneurysms. Br J Neurosurg.

[7] Meckel S, McAuliffe W, Fiorella D, Taschner CA, Phatouros C, Phillips TJ, Vasak P, Schumacher M, Klisch J (2013). Endovascular treatment of complex aneurysms at the vertebrobasilar junction with flow-diverting stents: initial experience. Neurosurgery.

[8] Monteith SJ, Tsimpas A, Dumont AS, Tjoumakaris S, Gonzalez LF, Rosenwasser RH, Jabbour P (2014). Endovascular treatment of fusiform cerebral aneurysms with the pipeline embolization device. J Neurosurg.

[9] Kulcsar Z, Ernemann U, Wetzel SG, Bock A, Goericke S, Panagiotopoulos V, Forsting M, Ruefenacht DA, Wanke I (2010). High-profile flow diverter (silk) implantation in the basilar artery: efficacy in the treatment of aneurysms and the role of the perforators. Stroke J Cereb Circ.

[10] McAuliffe W, Wenderoth JD (2012). Immediate and midterm results following treatment of recently ruptured intracranial aneurysms with the pipeline embolization device. AJNR Am J Neuroradiol.

[11] De Vries J, Boogaarts J, Van Norden A, Wakhloo AK (2013). New generation of Flow Diverter (surpass) for unruptured intracranial aneurysms: a prospective single-center study in 37 patients. Stroke J Cereb Circ.

[12] Siddiqui AH, Abla AA, Kan P, Dumont TM, Jahshan S, Britz GW, Hopkins LN, Levy EI (2012). Panacea or problem: flow diverters in the treatment of symptomatic large or giant fusiform vertebrobasilar aneurysms. J Neurosurg.

[13] Munich SA, Tan LA, Keigher KM, Chen M, Moftakhar R, Lopes DK (2014). The pipeline embolization device for the treatment of posterior circulation fusiform aneurysms: lessons learned at a single institution. J Neurosurg.

[14] Toth G, Bain M, Hussain MS, Moskowitz S, Masaryk T, Rasmussen P, Hui F (2015). Posterior circulation flow diversion: a single-center experience and literature review. J Neurointerventional Surg.

[15] Wakhloo AK, Lylyk P, de Vries J, Taschner C, Lundquist J, Biondi A, Hartmann M, Szikora I, Pierot L, Sakai N, Imamura H, Sourour N, Rennie I, Skalej M, Beuing O, Bonafe A, Mery F, Turjman F, Brouwer P, Boccardi E, Valvassori L, Derakhshani S, Litzenberg MW, Gounis MJ (2015). Surpass flow diverter in the treatment of intracranial aneurysms: a prospective multicenter study. AJNR Am J Neuroradiol.

[16] Arrese I, Sarabia R, Pintado R, Delgado-Rodriguez M (2013). Flow-diverter devices for intracranial aneurysms: systematic review and meta-analysis. Neurosurgery.

[17] McLaughlin N, McArthur DL, Martin NA (2013). Use of stent-assisted coil embolization for the treatment of wide-necked aneurysms: a systematic review. Surg Neurol Int.

[18] Shapiro M, Babb J, Becske T, Nelson PK (2008). Safety and efficacy of adjunctive balloon remodeling during endovascular treatment of intracranial aneurysms: a literature review. AJNR Am J Neuroradiol.

